# Free‐Volume Regulation Enables Significantly Enhanced Electrical Breakdown via Short‐Chain Molecular Spatial‐Positioning Intercalation into Poly(vinylidene fluoride)

**DOI:** 10.1002/advs.202517894

**Published:** 2025-11-18

**Authors:** Ziyue Wang, Jiyang Xie, Wanbiao Hu

**Affiliations:** ^1^ Yunnan Key Laboratory of Electromagnetic Materials and Devices National Center for International Research on Photoelectric and Energy Materials School of Materials and Energy Yunnan University Kunming 650091 P. R. China; ^2^ Electron Microscopy Center Yunnan University Kunming 650091 P. R. China; ^3^ Southwest United Graduate School Kunming 650092 P. R. China; ^4^ School of Engineering Yunnan University Kunming 650091 P. R. China

**Keywords:** breakdown strength, energy storage, free volume, PVDF film, spatial‐positioning

## Abstract

The peculiar molecule‐structural units with ferroelectric ordering render poly(vinylidene fluoride) (PVDF) a crucial functional polymer, but the existence of the free volume in PVDF (also for many other polymers) would interrupt the long‐range molecule chains, leading to the declined electrical functionality (e.g., electric breakdown), and also the thermal, mechanical, and chain relaxation properties. Herein, to regulate the free volume for electrical tuning, a molecular spatial‐positioning shimming strategy is developed in terms of intercalating shortchain molecules polyethylene wax (PE wax) into the spherulite gap region of PVDF. The PE wax/PVDF films are fabricated by a multi‐layer folding coupled hot pressing route that allows the complex crystalline structure modulation (e.g., phase, morphology, spherulite formation, amorphous and free‐volume status *etc*.), which are comprehensively investigated. Upon free volume regulation, the fabricated PE wax/PVDF film exhibits significantly improved breakdown strength (*E*_b_ = 735.1 MV m^−1^). Meanwhile, ultrahigh energy density (32.67 J cm^−3^) and energy storage efficiency (78.02%) are synergistically achieved. With ultrafast energy release (*t*_0.9_ = 38.5 ns) and exceptional power density (138 MW cm^−^^3^), the charge–discharge performance competes favorably with leading polymer/hybrid‐based films. This work offers a novel model to design new PVDF (or other polymer) based films with generating superior functionality.

## Graphical abstract

An innovative molecular spatial‐positioning shimming strategy is developed to optimize the electrical properties of poly(vinylidene fluoride) (PVDF). By intercalating polyethylene wax (PE wax) short‐chain molecules into PVDF's free volume, the film achieves a significantly enhanced energy density of 32.67 J cm^−^^3^.

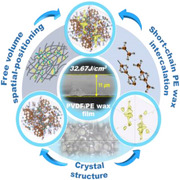

## 1 Introduction

**Figure 1:**
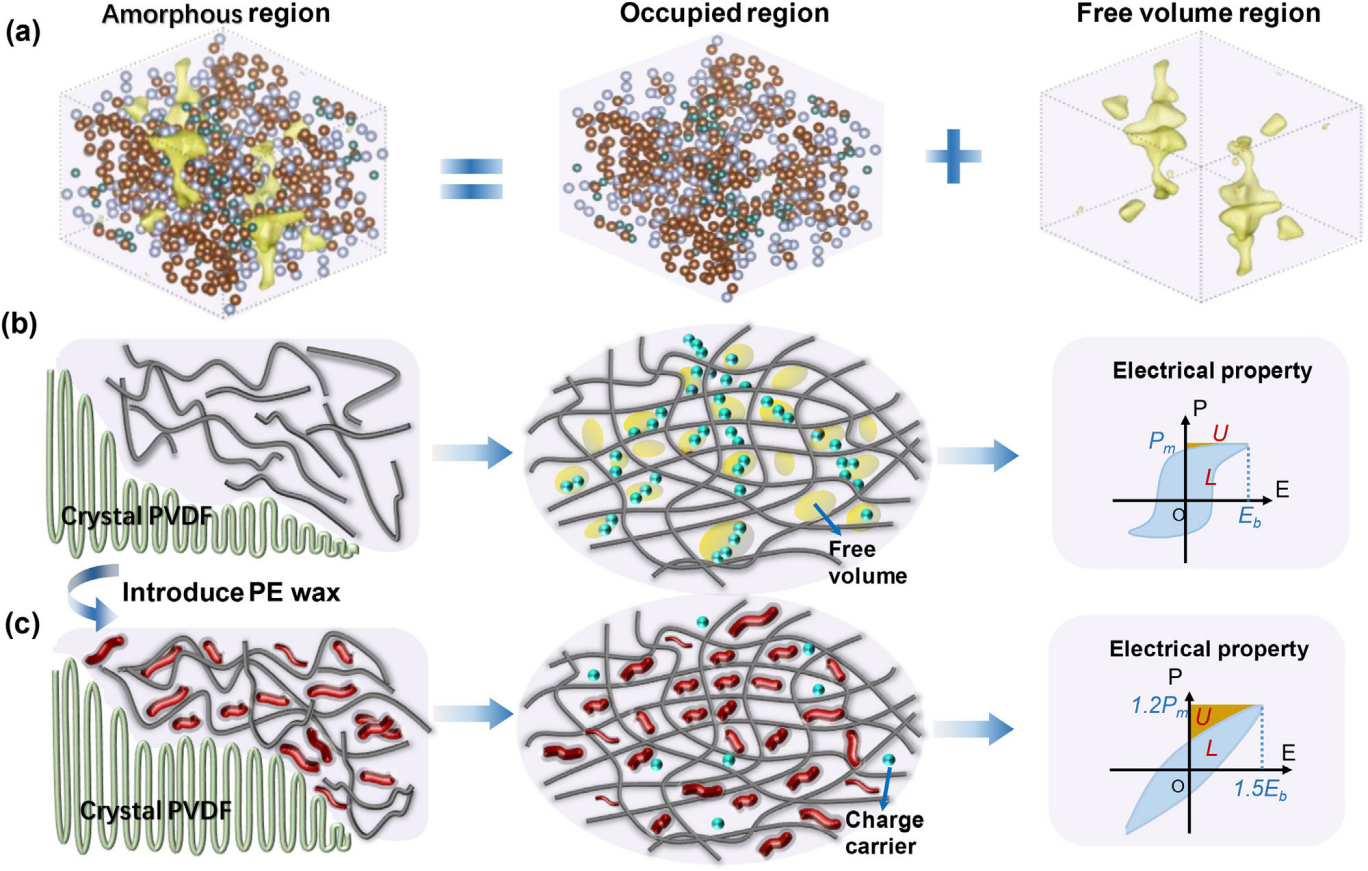
Proposed molecular spatial‐positioning shimming strategy. a) Schematic diagram of the free volume distribution of a certain polymer, *e.g*. PVDF here. b) Structure‐charge transport induced primitive electrical property in PVDF with free volume. c) Molecular (PE wax) spatial‐positioning shimming optimized structure‐charge dynamic for the enhanced electrical property.

PVDF‐based ferroelectric films, as a basic sort of multifunctional materials, exhibiting, e.g., excellent flexibility, electrical insulation, and withstanding voltage *etc*., find many important applications in power devices and energy storage systems.^[^ ^1^, ^2^, ^3^ ^]^ These advanced merits stem primarily from the special strong covalent interactions of the basic molecule‐structural units in variable polymerization degrees, which in turn influence the overall microstructures and macroproperties.^[^ ^4^, ^5^ ^]^ One prominent aspect involved in PVDF, which also differs from the crystalline inorganics with periodic arrangements of the rigid atoms, is the general coexistence of the crystalline regions and amorphous regions (depicted in **Figure** **1**). In contrast to crystalline regions, the amorphous regions exhibit ample unoccupied spaces between molecules, resulting in what is termed “free volume”, as shown in Figure 1a, which is constituted by various of small empty spaces or interstices between the molecules, resembling pores in form.^[^ ^6^, ^7^ ^]^

Although free volume in PVDF comprises only a minor fraction, e.g., ≈2.5 vol.%, it severely restricts molecular chain motion, degrading thermal, mechanical, and chain relaxation properties *etc*.^[^ ^4^, ^8^ ^]^ This explains why PVDFs experimental breakdown strength (400–500 MV m^−1^) falls far below its theoretical limit (>800 MV m^−1^).^[^ ^9^, ^10^, ^11^ ^]^ Similar discrepancies occur in most polymers due to free volume disrupting covalent interactions and charge dynamics. To this point, Wagner had, with presenting numerous micropores located between three spherulites in cross‐linked polyethylene, observed the discharge channels developed along the boundaries of these spherulites.^[^ ^12^ ^]^

According to the free volume breakdown theory, free electrons, propelled by electric field, amass higher energy levels. As indicated in Figure 1b, these electrons accelerate under external fields and attain sufficient energy levels within the hole possessing the longest free path, initiating the breakdown process. A similar process also has been monitored in polypropylene by Kolesov, who reported the deterioration in breakdown strength with increasing spherulite diameter, suggesting that breakdown occurs in the free volume of inter‐spherulite regions.^[^ ^13^ ^]^ As a consequence, whatever for PVDF or other more polymers, it falls into a predicament, up to date, that these behaviors and features rooting in the inherent structure of the polymers can not be effectively controlled and optimized.^[^ ^14^, ^15^ ^]^

To effectively limit carrier movement and inhibit conductivity losses for polymer property tuning, we devise a novel molecular spatial‐positioning strategy to precision‐engineer PVDF's free volume architecture using short‐chain polyethylene wax (PE wax). This attempt is coupled with the premise that the chemical crosslinking, multiple hydrogen bonding and grafting reactions between certain short‐chain molecules and polymers facilitate the construction of the carrier (electron) transport barriers within free volume region.^[^ ^16^, ^17^ ^]^ Accordingly, an ideal transmission barrier, polyethylene wax (PE wax) consisting of short‐chain molecular (molecular weight: M_n_ ≈ 2000–3000), is introduced into the amorphous region of PVDF. As shown in Figure 1c, PE wax does not penetrate the densely packed crystalline regions during PVDF co‐crystallization, but instead migrates preferentially into amorphous regions, achieving molecular spatial‐positioning shimming intercalation. The intercalated PE wax occupies a portion of the free volume to increase electron scattering probability for shortening the electron mean free path, and also creates additional obstacles for electron transport, consequently greatly improving the electrical insulation and mechanical breakdown. Through systematic microstructure and property investigations, the fabricated PE wax/PVDF film can exhibit a significantly enhanced electrical breakdown strength of 700 MV m^−1^ and comes along with the ultrahigh electrostatic energy density of 32.67 J cm^−3^. Beyond unprecedented charge–discharge power density (138 MW cm^−3^), the composite exhibits ultrafast discharge kinetics (*t*_0.9_ = 38.5 ns) and cyclability surpassing 10⁵ cycles, challenging the conventional energy‐power density trade‐off in dielectric polymers. This work establishes a new paradigm for molecular‐scale dielectric engineering, bridging the long‐standing gap between theoretical predictions and practical polymer dielectric performance.

## 2 Results and Discussion

### 2.1 Molecular Spatial‐Positioning Intercalation for Free‐Volume Regulation

Along with the proposed molecular spatial‐positioning intercalation strategy for fabricating the PE wax/PVDF films, a multi‐layer folding coupled hot pressing method is performed (details are seen in experimental section).^[^ ^18^ ^]^ Upon short‐chain PE wax intercalation into PVDF, complex crystalline structure (e.g., phase, morphology, spherulite formation, amorphous and free‐volume status *etc*.) modifications emerge due to stress‐free melt crystallization. Therefore, the involved structures are comprehensively investigated first.

**Figure 2:**
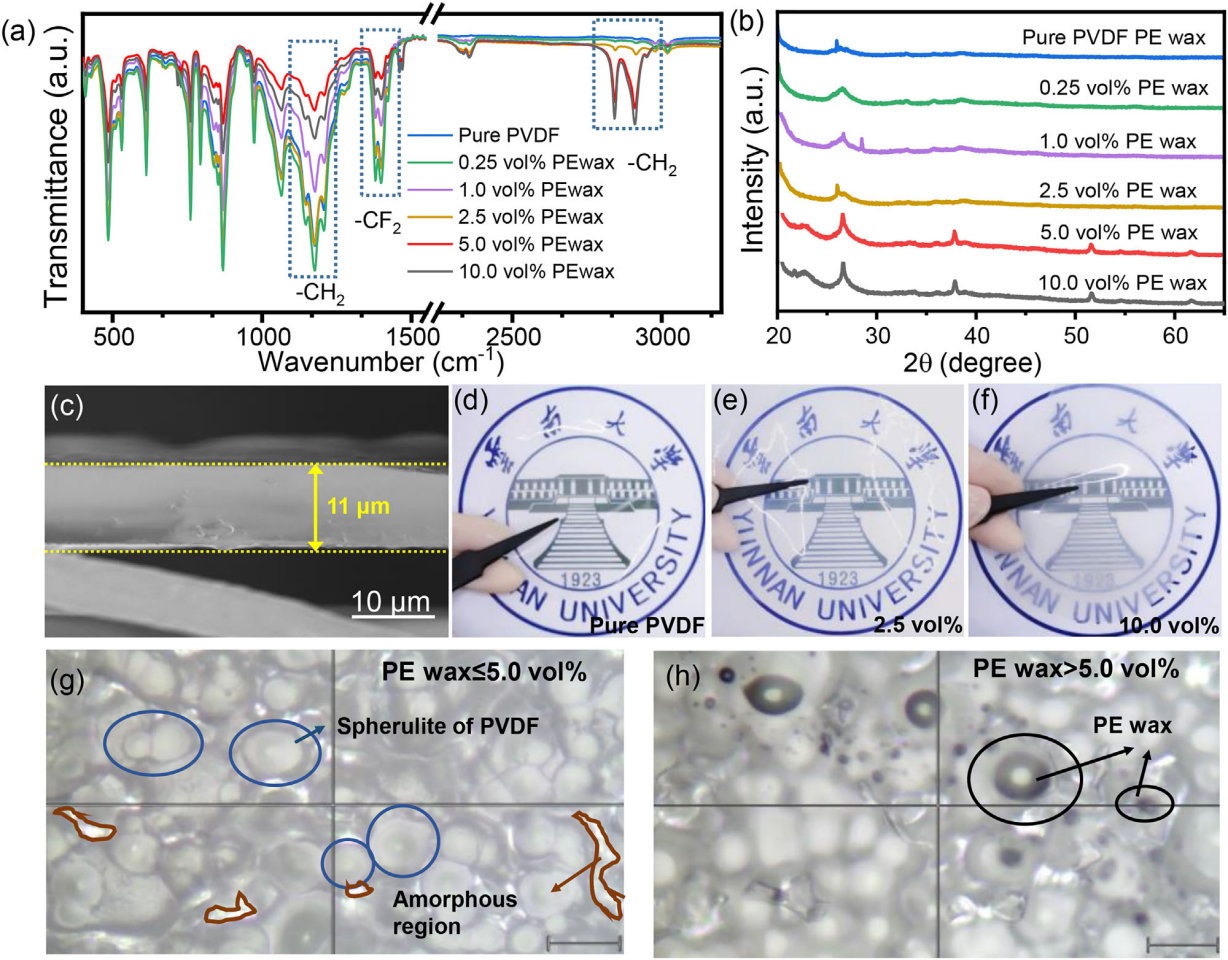
Structure characterizations of PE wax/PVDF films. a) FT‐IR spectra. b) XRD patterns. c) Cross‐sectional SEM image. d–f) photographic images for the translucency. g,h) Optical‐microscope visualizations on microstructures (crystalline/amorphous regions and intercalated PE wax). Scale bar: 20 µm.

To identify the PE‐wax molecular spatial‐positioning shimming for free‐volume regulation, delving deeper into the structural nuances of the PE wax/PVDF polymer blend, and meticulous analysis are conducted. Fourier Transform Infrared Spectroscopy (FT‐IR) and X‐Ray Diffraction (XRD) are employed to elucidate the intricate phase composition and crystal structure modifications triggered by the introduction of PE wax. FT‐IR spectra (**Figure** **2**a) depict the prominent absorption bands centered at 1176 and 1420 cm^−1^ that originate from the stretching vibrations of the CF_2_ group, indicating the characteristic chemical bonds within PVDF. The sharp peaks observed at 853, 795, and 623 cm^−1^ are associated with the vibrational modes of the crystalline phase, underscoring the crystalline nature of the PVDF backbone.^[^ ^19^, ^20^ ^]^ While, the absorption band at 550 cm^−1^ signifies the amorphous absorption characteristics, pointing to the presence of amorphous regions within the polymer blend.^[^ ^21^ ^]^ As the PE wax content increases, a notable enhancement in the CH_2_ absorption peak of PE becomes evident in the 2800–2900 cm^−1^ region.^[^ ^22^, ^23^ ^]^ This augmentation signifies the successful incorporation of PE wax into the PVDF matrix. Furthermore, the deformation vibration of the CH_2_ group, manifesting as an absorption band at 1210 cm^−1^, experiences a slight shift to a lower wavenumber due to its interaction with the CF_2_ group. This shift is indicative of the molecular spatial‐positioning shimming, reflecting the altered molecular arrangements and interactions within the PE wax/PVDF.

Complementing the FT‐IR analysis, XRD patterns (Figure 2b) reveal intricate details about the semi‐crystalline state of PE wax/PVDF, as evidenced by the unchanged intensity of the characteristic diffraction peaks (2θ = 26.5°) when the PE wax content increases from 0 to 10.0 vol%. This phenomenon suggests that the addition of PE wax does not affect the transition of the crystal phases (*α* and *β*) in the PVDF matrix and further underscores the compatibility and stability of PE wax within the gaps in amorphous regions of PVDF.^[^ ^24^ ^]^ Representative SEM micrograph (Figure 2c) display the cross‐section morphology of the PE wax/PVDF films. It is evident that PE wax, at concentrations up to 5.0 vol%, exhibits remarkable dispersibility within the PVDF matrix without any appreciable agglomeration. This homogeneous distribution effectively mitigates the formation of voids and imperfections in the composite films, as shown in Figure 2d–f, enhancing their overall structural integrity. The transparency remains unaffected even with a rise in the addition of 2.5 vol% PE, indicating that a low amount of PE wax does not infiltrate the crystalline region of PVDF and impact its degree of crystallinity.

With regard to the structural features for free‐volume regulation, although the inherent static volume existing between molecular chains in the amorphous regions is sub‐nanometric, its distribution profoundly impacts film properties. The unique existence of these free‐volume within the amorphous phase serves as a proxy for the determination of free volume, which, in turn, influences the overall performance of the polymer film. Although the inherent static free volume between the molecular chains exists in the amorphous region, the scale of free volume is generally so tiny that cannot be seen under a light microscope with magnitudes below the nanometer level. As a result, the free volume can be determined by observing the distribution of the amorphous/crystalline region since its unique existence in amorphous phase. By carefully analyzing the distribution of amorphous and crystalline regions, one can indirectly infer the scale and distribution of free volume within the PE wax/PVDF film.

Large‐scale polarized light microscopy image of the PE wax/PVDF film in Figure S1a (Supporting Information) reveals well‐defined spherulitic structures, as evidenced by the characteristic Maltese black cross pattern—a hallmark of radially symmetric crystalline lamellae within spherulites.^[^ ^25^ ^]^ Without polarized light, Figure 2g,h reveal a clearer view of the film's crystalline morphology and crystalline‐amorphous boundaries. Spherulites of varying sizes overlap, forming a dense stack, with brighter gaps due to the lower refractive index of the amorphous region. Based on the image, the film's crystallinity is estimated to be ≈70%. The introduction of PE wax does not significantly affect crystallinity, suggesting that PE wax remains in the amorphous region rather than penetrating the crystalline phase of PVDF. The excess PE wax (>5.0 vol%) underwent extraction and aggregation in the PVDF matrix, in frosting on the surface of the film in the form of small oil droplets, consistent with observed transparency shown in Figure 2d–f.

**Figure 3:**
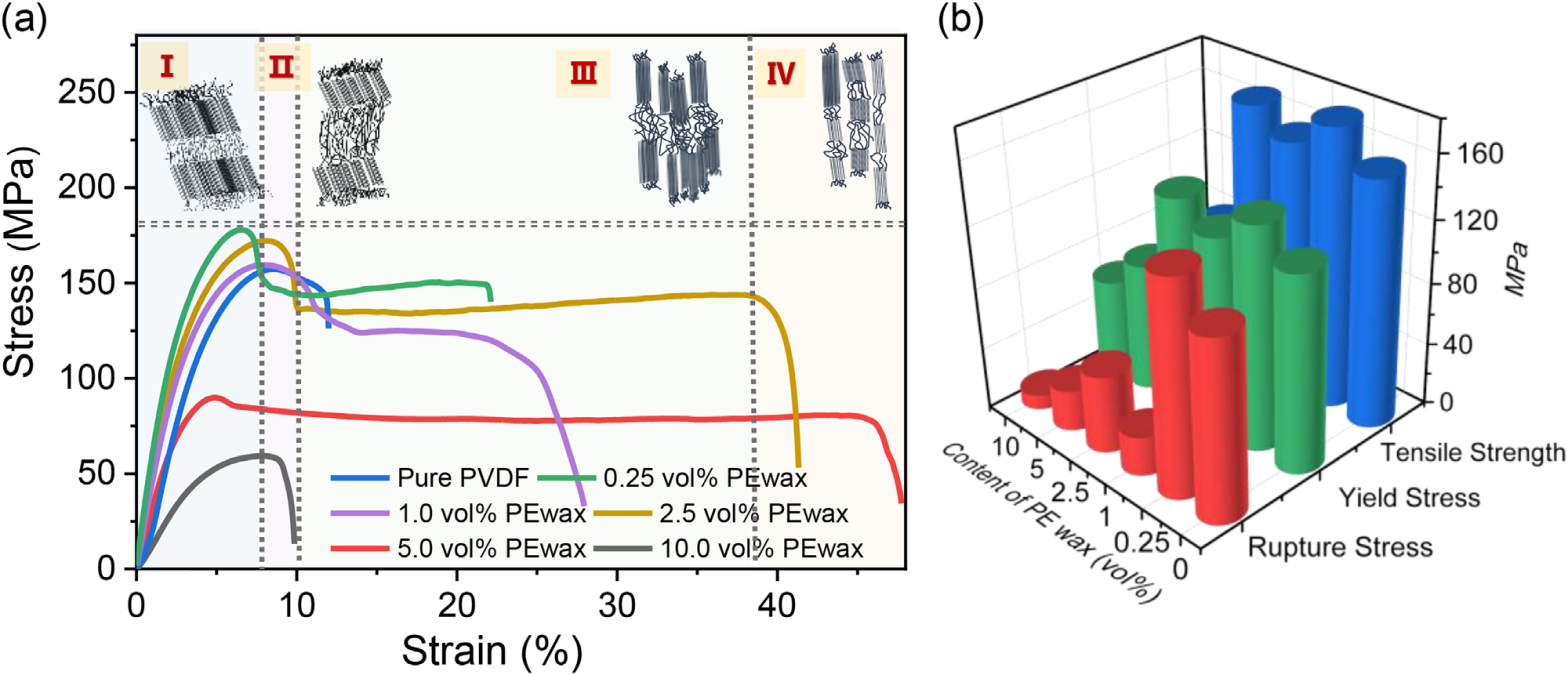
Structural evolutions through monitoring the mechanical changes of the of PE wax/PVDF films. a) Stress changes as a function of the strain. Insert shows molecular chain state changes in different four stages during stretching. b) Columnar diagram of mechanical properties with different PE wax contents.

To further gain the insight whether PE wax spatially intercalates into the non‐crystalline region of PVDF, the mechanical properties of the PE wax/PVDF films are investigated. The investigation is helpful in assessing molecular chain structure, rupture, and crystallization behaviors since different sizes of moving units respond differently to stress. **Figure** **3**a shows the deformation process during the stretching of crystalline PVDF films. It primarily involves the deformation of lamellae within spherulites that can be divided into four stages (Figure 3a‐insert): Stage‐I is characterized by the elastic deformation strictly following Hooke's law without plastic deformation. Stage‐II correlates with the phase transition and twining that are characterized by the tilting, sliding, and rotation of lamellae along the molecular axis. stage‐III involves lamellar rupture, where tilting, sliding, and rotation increase, causing some molecular chains to be pulled out from the crystallite. Finally, stage‐IV produces the microcrystalline structures composed of ruptured and elongated chains. Lamellar slip with crystallographic deflection along the molecular axis leads to thinning and elongation of lamellae.

During the elastic deformation (stage‐I), which involves strains below 5.0 vol%, the slope of the stress‐strain curve represents the intrinsic cohesive energy density of the film, reflecting the strength of intermolecular bonding. When the amount of added PE wax is lower than the free volume fraction of PVDF, the elastic modulus remains unchanged. This supports the findings of the spectroscopic and spectrometric tests mentioned earlier, and indicates that PE wax does not infiltrate the densely packed crystalline region of PVDF. The stretching in stage‐II corresponds to forced high elastic deformation, where the stress‐strain curve reaches a maximum point known as the yield point. As stress continues to increase, it becomes sufficient to overcome the energy barriers required for chain motion resulting in forced chain scission. At this stage, the short‐chain molecules of PE wax that enter the amorphous free volume act as plasticizers, facilitating the slip of PVDF molecular chains at the ends and transforming the film's mechanical properties from stiff and strong to soft and tough. This transition represents a shift to the viscous flow state, accompanied by the sliding of entire molecular chains. However, if the amount of added PE wax exceeds the free volume fraction, particularly at a filling level of 10.0 vol%, the excess PE wax becomes an insulating weak point under stress concentration that cause chain rupture and resulting in a film with soft and weak mechanical characteristics. Consequently, the fracture elongation also decreases, as depicted in Figure 3b. Modulus reduction (Figure S2, Supporting Information)correlates with PE wax aggregation, which introduces compliance and defects in the amorphous matrix, consistent with mechanical failure modes observed in tensile tests (Figure 3b)

**Figure 4:**
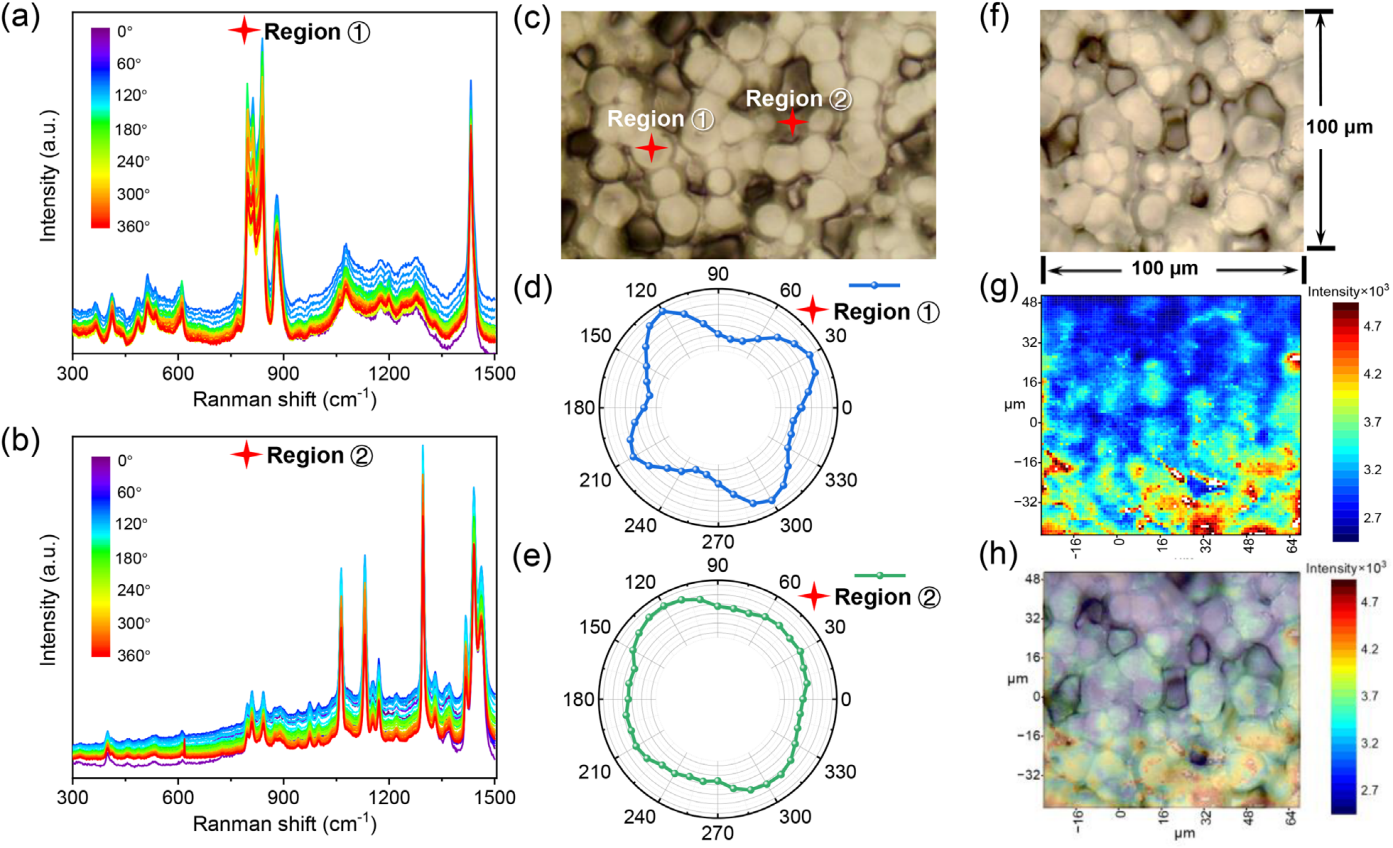
Spatially Resolved Molecular Orientation and Phase Distribution in PE Wax/PVDF Films. a) Polarized Raman spectra (0/360° polarization angles, 10°/step) acquired from the spherulite core (Region **①**) and b) the inter‐spherulitic gap (Region **②**). Schematic of the angle‐resolved polarized Raman spectroscopy (c) Optical micrograph demarcating Regions **①** (spherulite core) and **②** (inter‐spherulitic gap). d) Polar plot of Raman intensity for Region **①**. e) Polar plot for Region **②**. f) Corresponding optical micrograph of in situ Raman mapping image. g) Raman intensity map of the 840 cm^−1^ PVDF peak (red: high intensity; blue: low). h) Overlay of (f) and (g), confirming PE wax localization in low‐crystallinity inter‐spherulitic regions.

To further elucidate the spatial distribution of PE wax within the PVDF and its confinement to the amorphous regions, polarized Raman spectroscopy and Raman mapping were employed to correlate molecular symmetry and phase‐specific interactions. **Figure** **4**a,b presents polarized Raman spectra acquired from two distinct regions of the PVDF spherulite: the spherulite core (Region ①, Figure 4c) and the inter‐spherulitic gap (Region ②, Figure 4c). The spectra from Region ① (Figure 4a) exhibit characteristic PVDF vibrational modes, including the CF_2_ symmetric stretching at 840 cm^−1^ and CH_2_ rocking at 796 cm^−1^, confirming the crystalline α/β‐phase coexistence. Critically, the absence of the 840 cm^−1^ PE wax signature in Region I unambiguously demonstrates that PE wax does not penetrate the densely packed crystalline lamellae of PVDF. In contrast, Region ② (Figure 4b) reveals a distinct peak at 840 cm^−1^, corresponding to the CH_2_ rocking mode of PE wax, alongside attenuated PVDF signals. This stark contrast confirms that PE wax is exclusively localized in the inter‐spherulitic amorphous regions rather than the crystalline cores, consistent with the proposed molecular spatial‐positioning shimming mechanism.

The polarized Raman polar plots (Figure 4d,e) provide deeper insights into molecular symmetry and chain alignment. For Region I (spherulite core, Figure 4d), the polar plot displays a pronounced four‐petal (quadrant‐symmetric) pattern, characteristic of the highly ordered β‐phase PVDF with all‐trans (TTT) chain conformation. This symmetry arises from the anisotropic polarizability of the aligned CF_2_ dipoles, which exhibit maximal Raman intensity when the laser polarization aligns with the chain axis. The sharp petals reflect enhanced spontaneous polarization due to coherent dipole alignment within the crystalline lamellae. In contrast, the polar plot for Region ② (Figure 4e) exhibits a smoothed, quasi‐isotropic loop with residual quadrant asymmetry. This hybrid behavior stems from the amorphous nature of the inter‐spherulitic regions, where PVDF chains adopt random coils with partial gauche conformations. The residual asymmetry in Region II arises from localized chain segments influenced by adjacent crystalline domains, while the overall isotropy reflects the absence of long‐range order. The presence of PE wax in these regions further disrupts chain alignment, as evidenced by the reduced intensity modulation in Figure 4e compared to Figure 4c. Crucially, the absence of a PE wax‐specific polar plot symmetry (*e.g*., isotropic or hexagonal) confirms that PE wax does not form crystalline aggregates but remains molecularly dispersed within the amorphous matrix.

Raman mapping (Figure 4f‐h) spatially resolves the distribution of PVDF's crystalline phase and PE wax. Figure 4f maps the intensity of the 840 cm^−1^ PVDF peak (CF_2_ symmetric stretching) across a 100 × 100 µm^2^ area (step size: 1 µm), with higher intensities (red regions) corresponding to crystalline spherulite cores. The optical micrograph (Figure 4f) reveals spherulite boundaries and inter‐spherulitic gaps, which align precisely with low‐intensity (blue) regions in Figure 4g. Overlaying these images (Figure 4h) confirms that the 840 cm^−1^ signal is maximized within spherulites and minimized in the inter‐spherulitic regions. This inverse correlation validates that PE wax occupies the amorphous inter‐spherulitic gaps, where PVDF crystallinity is inherently low. The homogeneous intensity distribution within spherulites (Figure 4f) further corroborates that PE wax does not infiltrate crystalline domains, as any disruption to the crystalline lattice would induce local intensity variations.

These findings collectively establish that PE wax is spatially confined to the amorphous regions, where it occupies free‐volume voids without perturbing PVDF's crystalline architecture. The polarized Raman data further reveal that PE wax disrupts local chain dynamics in the amorphous matrix, reducing orientational order while preserving crystalline phase integrity. This structural refinement aligns with the enhanced electrical breakdown strength and energy storage performance, as the amorphous‐phase modifications suppress charge carrier mobility without compromising the polarization‐active crystalline regions.

In summary, the evaluation of the mechanical properties and Raman phase distribution of PE wax/PVDF films supports the model of PE wax filling into the free volume, establishing a consistent structure‐activity relationship for subsequent dielectric and energy storage behavior with high electric field.

### 2.2 Effect of Free Volume Regulation on Dielectric and Relaxation

**Figure 5:**
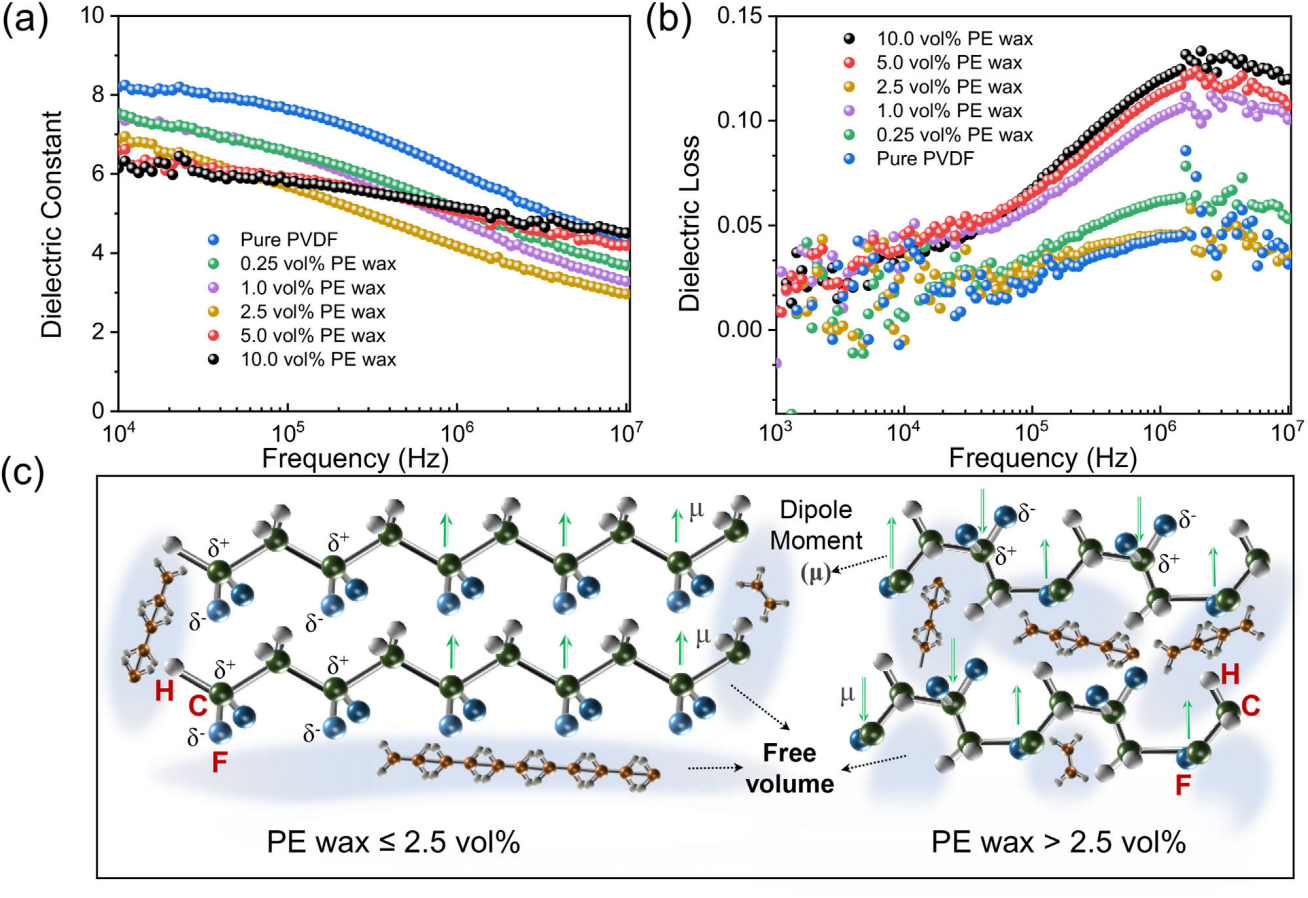
Dielectric behaviors of PE wax/PVDF films. Frequency dependence of a) dielectric constant and b) dielectric loss. c) Effect of PE wax content on dipole orientation and relaxation dynamics in PE wax/PVDF film.

The free volume in PVDF undergoes dynamic reorganization as the polymer chains transition into a viscoelastic state under applied electric fields, significantly altering its dielectric response.^[^ ^26^ ^]^ The dielectric properties of the PE wax/PVDF films are presented in **Figure** **5**. As shown in Figure 5a, the dielectric constant decreases with increasing PE wax content, which arises from two interrelated mechanisms governing dipole reorientation and chain dynamics. In polar PVDF molecules, the orientation of the permanent dipole depends on the local environment in which it resides. The ability of the dipole to orient itself is affected by whether it is located in an amorphous or crystalline region. Reducing the free volume can enhance the restrictions imposed by the crystalline region. First, when the PE wax content is below the free volume fraction (2.5 vol%), the PE wax fills the pores in the amorphous regions and decreases the average free path length (mean square end‐to‐end distance, $\bar{x}$) of the polymer chains. As depicted in Figure 5c, a reduced end‐to‐end distance implies that the inter‐chain interactions increase, resulting in enhanced intermolecular forces and viscosity within the PVDF chains.^[^ ^27^ ^]^ The ability of permanent dipoles to reorient themselves in response to an applied electric field diminishes, thereby lowering the polarization capability of the material and, consequently, its dielectric constant. When PE wax is incorporated, it not only fills the voids but also modifies the potential energy landscape that the PVDF chains experience. As more PE wax is added, the free volume continues to decrease, exerting less delocalized stress on the PVDF chains. This compression disrupts the arrangement of the chains and makes it easier to adopt bent geometries which is energetically favorable but may compromise their polarization properties. Specifically, the compressive forces on the PVDF polymer chains lead to conformational changes from the trans configuration to the gauche and cis forms. Trans to gauche/cis transition supports effective dipole alignment, gradually converts to the gauche conformation under increasing stress. This transition decreases the dipole moment in each unit cell, which in turn affects the overall dielectric constant.^[^ ^28^ ^]^ Second, PE wax is a non‐polar polymer with a symmetrical and identical structure possess a net sum of bond dipole vectors equaling zero (*q* = 0) and a low dielectric constant (*ε*≈2‐3). Upon incorporating the PE wax into the PVDF matrix, a notable reduction in the overall dielectric constant of the system was observed. In particular, as excess short‐chain PE wax molecules occupy the available space and subsequent overflow into the PVDF matrix facilitates a synergistic effect, certain long‐chain PVDF molecules are enabled to attain sufficient plasticity for more significant dielectric relaxation. This phenomenon enhances the alignment of electric dipoles parallel to the applied electric field.

Furthermore, at lower filling amounts, the dielectric loss initially rises and then down with an increase in the PE wax content, as depicted in Figure 5b (PE wax≤2.5 vol%), can be explained by considering the impact of PE wax on the molecular dynamics and intermolecular interactions within the composite film. The restricted chain movement leads to diminished dipole reorientation capabilities, causing an initial increase in dielectric loss due to the reduced mobility of the dipoles. Although the free volume decreases as PE wax occupies space in the polymer matrix, the overall mobility of the polymer chains is still relatively high. As the PE wax content approaches a critical level, the more organized crystalline regions enhance local order, leading to a return of dielectric loss as the chains in the more rigid regions become less responsive to external electric fields. However, at higher filling amounts, excess PE wax may induce phase separation, leading to heterogeneous structural domains that can have varying dielectric responses. New dipole losses are introduced since the energy dissipation associated with dipolar relaxation phenomena increases, resulting in a rapid increase in dielectric loss. This can be seen from the change of dielectric constant of excess PE wax system (Figure 5a, PE wax> 2.5 vol%), and its response ability to high frequency electric field is reduced. Due to the presence of wax, the chain responds slowly to the applied field, causing frequency‐dependent loss tangents to escalate at higher frequency. In summary, the overall dielectric behavior of the PE wax/PVDF composite films can be elucidated by the combined effects of increased intermolecular forces and viscosity at lower filling amounts of PE wax, as well as the low dielectric constant and introduction of new dipole losses at higher filling amounts.

### 2.3 Improved Electric Breakdown for Ultrahigh Electrostatic Energy Storage

**Figure 6:**
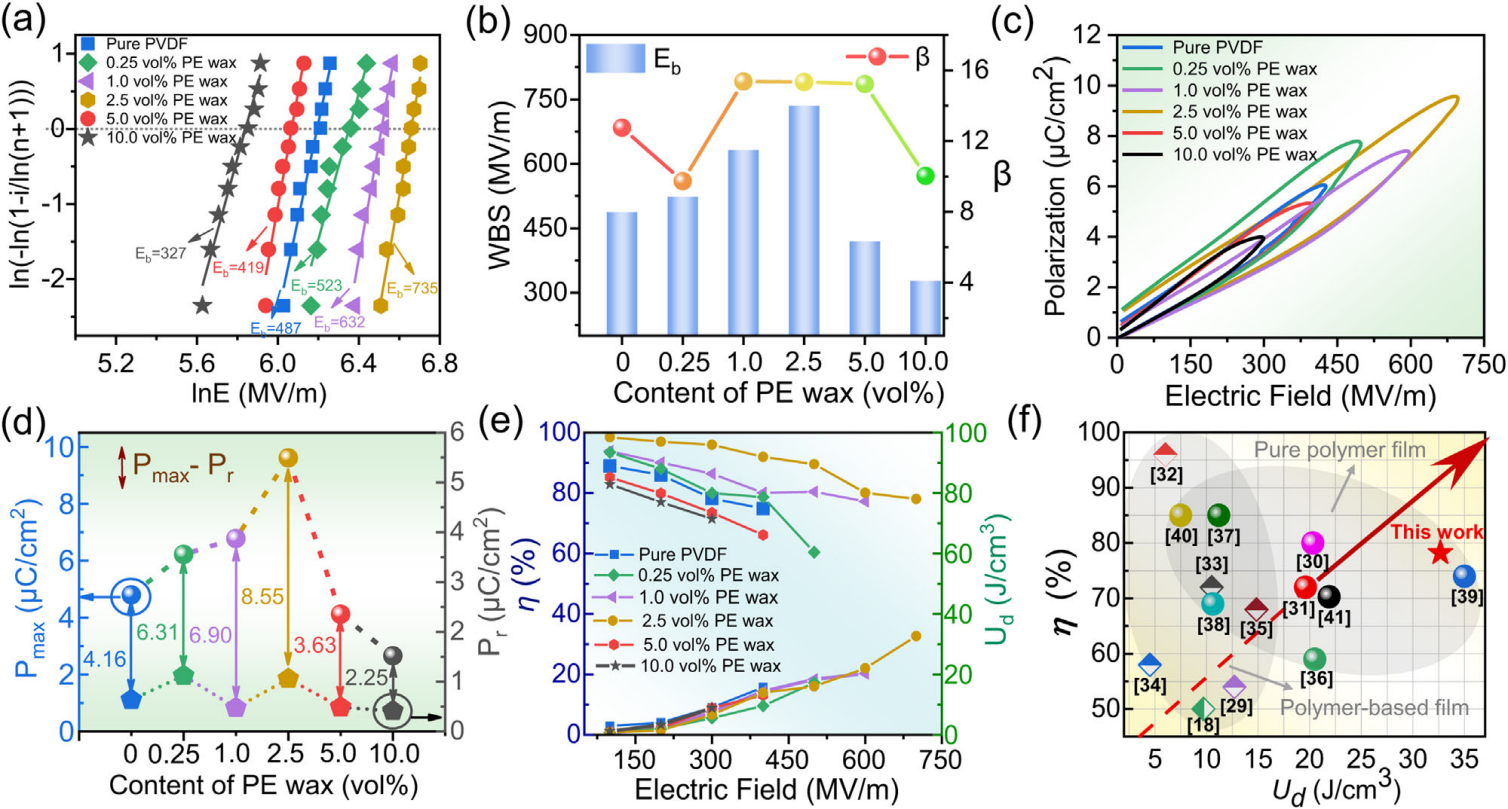
Electric breakdown and energy storage properties of PE wax/PVDF films. a) Weibull distributions. b) Breakdown strength (*E*_b_) and shape parameters (*β*) deduced from Weibull distributions. c) Unipolar P‐E loops. d) Maximum polarization (*P*_max_), residual polarization (*P_r_*), and their differences (*P*_max_‐*P*_r_) deduced from P‐E loops. e) Discharge density (*U*_d_) and energy efficiency (*η*) as a function of the applied electric fields. f) Comparison of *U*_d_ and *η* with some well‐known reported works, mainly the PVDF‐based organic/inorganic composite films and all‐organic polymer films.

As many functions and applications, especially the electricity, are associated with the charge transportation, the electrical properties are evaluated. **Figure** **6**a shows the breakdown characteristics of PE wax/PVDF dielectric films. The statistical description of breakdown behavior is commonly encapsulated in a two‐parameter Weibull distribution, encompassing both the breakdown strength (*E*_b_) and shape parameters (*β*).^[^ ^29^, ^30^ ^]^ It reveals a noteworthy trend: as the content of PE wax increases, the breakdown strength augments until a critical threshold is reached, approximately corresponding to the free volume of PVDF. Notably, the highest breakdown strength of 735.1 MV m^−1^ is attained at a PE wax additive content of 2.5 vol%. This observation further elucidates the mechanistic intricacies of breakdown initiation and propagation within the free‐volume regions. When a voltage is applied, electrons are injected into the PVDF matrix through the surface electrode, resulting in a supralinear increase in both the density and drift velocity of charge carriers.In this scenario, the emergence of low‐density regions (free volume) act as hotspots for electron acceleration, leading to collision ionization and subsequent chain scission and dielectric breakdown. Beyond the direct contribution of charge carriers, the free‐volume pores themselves gradually expand under the electric field, forming localized insulation flaw clusters. These clusters eventually coalesce into discrete breakdown channels aligned with the electric field direction. The infusion of PE wax into the free volume effectively suppresses the collision ionization and secondary electron emission events that occur within these channels, while simultaneously increasing electron scattering events. Together, these effects enhance breakdown resistance to a certain extent. However, However, when the PE wax content exceeds the free‐volume capacity of PVDF, the excess short‐chain PE molecules act as defects in the insulation system. These surplus molecules tend to agglomerate into oil‐like droplets (Figure 2h), forming conductive pathways that facilitate charge transport and degrade the film's insulating properties. This occurs because PE wax molecules, with their smaller size and less consolidated structure compared to PVDF, exhibit lower intrinsic resistance to electrical breakdown when aggregated. By analogy to the charge transport pathways illustrated in Figure 1c, these agglomerated regions enable charge percolation across the PVDF matrix, akin to a percolation effect. Consequently, the breakdown strength rapidly diminishes and may fall below the intrinsic breakdown strength of pure PVDF. Regarding reliability, the average Weibull modulus (*β*) for most samples exceeds 9 (Figure 6b), indicating high reproducibility in breakdown testing. To further unravel the breakdown mechanisms and their correlation with experimental observations, a quantitative analysis of free‐volume regulation and short‐chain molecular intercalation is imperative.

To delve deeper into the breakdown mechanisms and their correlation with experimental phenomena, it is imperative to quantitatively analyze the roles of free‐volume regulation and short‐chain molecular spatial‐positioning intercalation. Merely focusing on the avalanche breakdown process falls short of experimental phenomena, such as the such as the intricate relationship between breakdown strength and PE wax content, thus the statistical or cumulative characteristics of breakdown should also to be considered. To address this, one can quantify the probability of electron hopping to the next trap behind the energy barrier as *P*_µ_. To describe this field‐dependent behavior quantitatively under high electric fields, a modified Poole‐Frenkel conduction model^[^ ^8^, ^31^ ^]^ can be employed:

(1) $$P_{\mu }=exp\left[-\left(E_{\mu }-eFx\right)/\left(kT\right)\right]$$

where *k* is the Boltzmann constant, *E*_μ_ is the breakdown barrier, *F* is the electric field. When subjected to an electric field, the electrons experience acceleration due to the force *eF*, acquiring a net drift velocity. These electrons then drift along the direction of the applied force (*eF*). The mean drift velocity is influenced by the height of the energy barrier between traps. If we denote the distance, as *x* (where the electrons are accelerated in the direction of *eF*), to the peak of the subsequent barrier, the height of the barrier *E*_μ_ will be reduced by *eFx*. This reduction corresponds to the energy gained by electrons over the displacement *x*. Thus, a shorter electron mean free path x (i.e., the characteristic free‐volume pore size) corresponds to a lower effective breakdown barrier. Consider the case of electron‐type breakdown:^[^ ^31^ ^]^

(2) $$\;E_{b}=\frac{E_{\mu }}{ex}$$

where *E*_b_ is the breakdown strength of the film, *e* = 1.6 × 10^−19^ C. It can be derived from the above two Equations (1) and (2) that breakdown initiation occurs when electrons gain sufficient energy via acceleration over the largest mean free path *x* within free‐volume pores, enabling them to surmount the barrier *E*_µ_. The intercalation of PE wax precisely shortens the mean free path of electrons and increases the breakdown barrier, thus increasing the breakdown strength (*E_b_*). The intercalation of short‐chain molecules plays a pivotal role in modifying the spatial positioning of the polymeric matrix. This not only tailors local free‐volume architecture but also alters electron transport dynamics. By altering the intermolecular spacing and interactions, short‐chain molecules reshape the energy landscape for electron migration, optimizing the breakdown behavior of the PE wax/PVDF film.

In general, the enhanced breakdown strength delays polarization saturation the dielectric films. Therefore, it also brings the improvement of energy storage density to a large extent. Unipolar P‐E loops measured at 100 Hz, as shown in Figure 6c, demonstrate once the addition of PE wax to the PVDF matrix up to a 2.5 vol% amount (close to the free volume of PVDF), can lead to a simultaneous enhancement in both breakdown strength and polarization of the PE wax/PVDF films. In this case, the highest energy density achieved at 32.67 J cm^−3^ while maintaining optimal energy efficiency at 78.02%. More P‐E loops of filled PE wax close to the free volume fraction are shown in Figure S3 and Table S1 (Supporting information). Under high fields, thermally activated neutral sites ionize, releasing free electrons. Electrons traversing free‐volume regions collide with PE wax molecules, losing kinetic energy via scattering. This process acts as a mechanism for scattering charge carriers, significantly reducing their mobility and energy. Such a microscale dynamic process has a macroscopic effect on the energy storage performance of the film. It not only increases the breakdown strength but also slows down the rate at which the film reaches polarization saturation, thereby enhancing the net polarization rate (*P*_max_‐*P*_r_), shown in Figure 6d. The discharged energy density and efficiency of PE wax/PVDF film are calculated based on the P‐E loops above. As clearly presented in Figure 6e, the highest *U*_d_ for PVDF filled with 0.25, 1.0, 2.5, 5.0, and 10.0 vol% of PE wax are 1.11, 1.31, 2.14, 0.88, and 0.60 times that of pristine PVDF (15.3 J cm^−3^), respectively.

All the results are favorable to point out that suppressing free volume breakdown can effectively improve the energy storage performance of PVDF‐based film. Figure 6f compares the energy density *U*_d_ and discharge efficiency *η* of this work with those of recently reported PVDF‐based dielectric films.^[^ ^18^, ^29^, ^32^, ^33^, ^34^, ^35^, ^36^, ^37^, ^38^, ^39^, ^40^, ^41^ ^]^ The integration of PE wax into the PVDF matrix, combined with the techniques of free‐volume regulation and short‐chain molecular spatial‐positioning intercalation, has revolutionized the landscape of polymer‐based energy storage dielectrics. In contrast, pure PVDF or other PVDF‐based organic/inorganic composites often suffer from limited energy density due to their inherent molecular structure and charge transport properties. The PE wax/PVDF system, modified through charge transport path optimization, demonstrates significant potential for controlling free volume in polymer‐based energy storage films.

**Figure 7:**
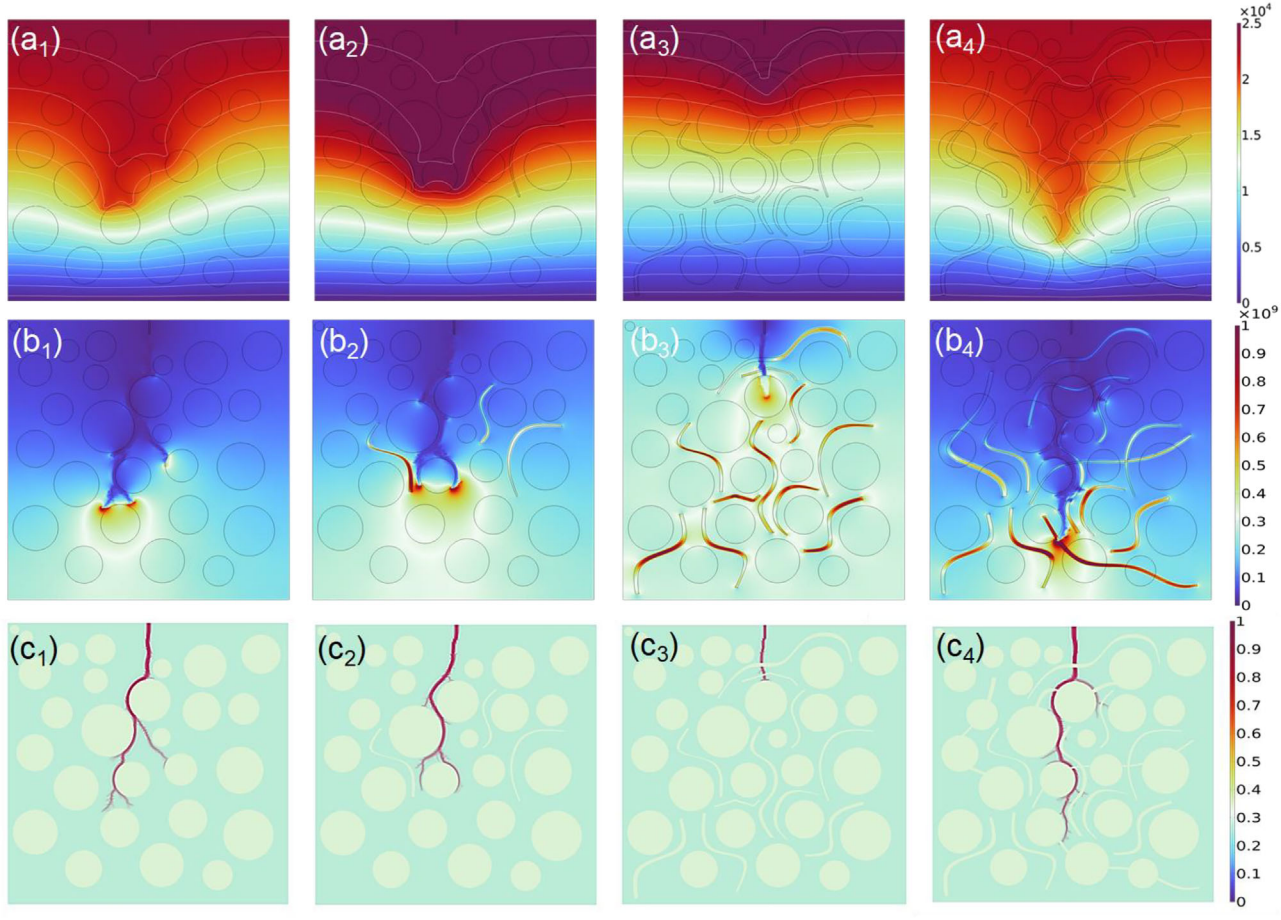
Simulation of the PE wax content evolution (0/0.25/2.5/5.0 vol%) in applied electric field. a_1_‐a_2_) The electric potential distribution. b_1_–b_3_) The electric field distribution. c_1_–c_3_) The spread of the current density of PVDF film (the trigger time of the applied field is 1.5 ns).

Finite element simulations (**Figure** **7**) provides a critical visualization of the dynamic interplay between free‐volume regulation, electric field redistribution, and electrical tree suppression in PE wax‐intercalated PVDF films. The electric potential maps (Figure 7 a_1_–a_4_) reveal how PE wax intercalation reshapes the electrostatic landscape of PVDF. In pure PVDF, the potential distribution exhibits pronounced inhomogeneity, accumulating space charges that distort the electric field. In contrast, the 2.5 vol% PE wax/PVDF (Figure a_3_) demonstrates a markedly uniform potential profile. The short‐chain PE wax molecules, fill the subnanometric free‐volume voids. The elimination of sharp potential gradients directly correlates with the suppression of charge injection at electrode interfaces, a critical factor in delaying breakdown initiation. The electric field simulations (Figure 7b_1_–b_4_) highlight the role of free‐volume regulation in suppressing field enhancement, a key driver of avalanche breakdown. In pure PVDF, the field distribution is highly nonuniform, with peak field intensities localized at spherulite boundaries and amorphous‐crystalline interfaces. The intercalation of PE wax into inter‐spherulitic gaps eliminates sharp field discontinuities, which otherwise act as field amplification sites. The temporal evolution of electrical treeing (Figure 7c_1_–c_4_) offers a dynamic perspective on how free‐volume regulation impedes breakdown progression. The electrical tree propagates rapidly along spherulite boundaries and amorphous channels, forming dendritic structures within 1.5 ns. This accelerated growth reflects the low‐energy pathway provided by free‐volume, where electrons gain sufficient kinetic energy to dissociate polymer chains via impact ionization. The intercalated PE wax molecules act as nanoscale barriers, disrupting the continuity of free‐volume pathways and forcing the tree to adopt a tortuous trajectory. This obstruction increases the effective ionization potential required for tree advancement, as electrons undergo frequent scattering with PE wax molecules. Consequently, the tree's propagation velocity decreases, delaying the percolation threshold and extending the film's lifetime under high fields. Other detail intial/transient/steady simulations are supported by Figures S4–S6 (Supporting Information). The simulations thus provide a spatial resolution of the breakdown strength enhancement, attributing it to the suppression of field‐driven electron acceleration in homogenized amorphous regions.

### 2.4 Ultrafast Charge–Discharge Dynamics and Record‐High Power Density for Advanced Capacitive Energy Storage

**Figure 8:**
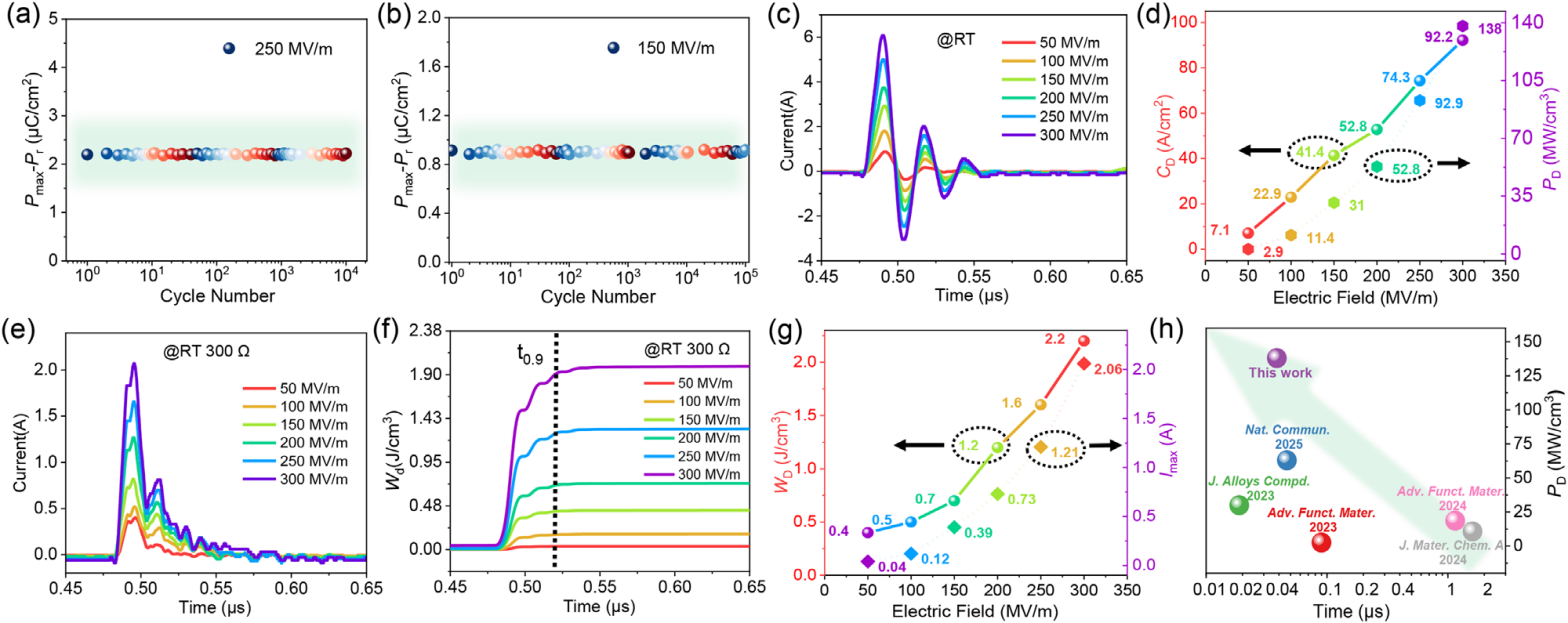
Charge–discharge performance in PE wax(2.5 vol%)/PVDF composite films. a) Cyclic stability under 250 MV m^−1^ (10 000 cycles). b) Ultralong cyclic stability under 150 MV m^−1^ (100000 cycles). c) Underdamped charge–discharge current‐time curves: transient current responses under 50–300 MV m^−1^ field strengths. d) Current density and power density derived from (c). e) Overdamped charge–discharge current‐time curves: current decay dynamics under 50–300 MV m^−1^. f) Energy density evolution integrated from (e). g) Field‐dependent energy density and peak current. h) Comparison of power density and charging speed with recent well‐known high‐performance dielectric films.

The PE wax(2.5 vol%)/PVDF film exhibits exceptional cyclic stability under high electric fields. At 250 MV m^−1^, the effective polarization (*P*_max_‐*P*_r_) remains ≈2.25 µC cm^−2^ after 10 000 cycles (**Figure** **8**a), and even after 100 000 cycles, the polarization loss is still stable (Figure 8b). This minimal degradation is attributed to the molecular spatial‐positioning intercalation of PE wax, which stabilizes the amorphous regions by reducing free‐volume‐induced chain slippage and interfacial charge trapping. The PE wax acts as a plasticizer in the amorphous matrix, mitigating mechanical fatigue while maintaining covalent interactions within crystalline PVDF, thereby preserving long‐range dipole alignment and minimizing dielectric hysteresis. The consistency between polarization loss and PE test values further validates the interfacial stability achieved through free‐volume regulation. Underdamped current‐time profiles (Figure 8c) reveal transient oscillations characteristic of LCR circuits, with peak current (*I*_max_) scaling linearly from 0.4 A (50 MV m^−1^) to 6.1 A (300 MV m^−1^). This behavior aligns with the classical damped harmonic oscillator model, where the damping coefficient (ζ) decreases with increasing field strength due to enhanced charge carrier mobility. The derived current density(*C*_D_) and power density(*P*_D_) (Figure 8d), can be calculated according to the formula:

(3) $$C_{D}=I_{max}/S$$

(4) $$P_{D}=EI_{max}/2S$$

exhibit a superlinear relationship with field strength, reaching 92.2 A cm^−^^2^ and 138 MW cm^−^^3^ at 300 MV m^−1^. Such high power density stems from the optimized free‐volume architecture, as described by the modified Poole‐Frenkel conduction model. The absence of negative differential resistance (NDR) at high fields confirms suppressed space charge accumulation, a critical factor for high‐rate energy delivery. In overdamped mode (Figure 8e), the current decays exponentially with a characteristic time constant t_0.9_ of 38.5 ns, indicating rapid energy release kinetics. The energy density evolution (Figure 8f) demonstrates near‐instantaneous charging, achieving 90% energy storage within 40 ns. The maximum energy density, can be calculated by:^[^ ^42^ ^]^

(5) $$W_{D}=R\int I\left(t^{2}\right)dt/V$$

scales quadratically with field strength, reaching 2.2 J cm^−^^3^ at 300 MV m^−1^, while *I*_max_ follows a linear trend (2.06 A at 300 MV m^−1^). This divergence highlights the dominance of capacitive energy storage over resistive losses, a hallmark of high‐quality dielectric film. Remarkably, as demonstrated in Figure 8h, the composite outperforms recent dielectric systems in both power density and charging speed compared to other well‐known work in recent years.^[^ ^43^, ^44^, ^45^, ^46^, ^47^ ^]^

## 3 Conclusion

In this contribution, a novel concept introduces a ground breaking strategy to boost the energy storage properties of PVDF‐based film. Central to this approach is the recognition of the pivotal role played by free volume within PVDF, serving as a determinant factor in governing its breakdown behavior. By implementing a molecular spatial‐positioning strategy through PE wax intercalation, we achieve precise regulation of PVDF's amorphous free volume architecture, effectively suppressing electron acceleration paths while preserving ferroelectric polarization. The resultant 2.5 vol% PE wax/PVDF composite demonstrates breakthrough energy storage characteristics: a record‐high breakdown strength of 735.1 MV m^−1^ synergistically drives an ultrahigh discharged energy density of 32.67 J cm^−^^3^ coupled with exceptional efficiency (78.02%), approaching the theoretical limit of PVDF‐based dielectrics. Crucially, the composite simultaneously achieves ultrafast discharge kinetics (38.5 ns, *t*_0.9_) and extraordinary power density (138 MW cm^−^^3^). These findings offer valuable insights into the design of polymer dielectric films optimized for high‐performance applications, particularly those leverage considerations of free volume to achieve superior electrical properties.

## 4 Experimental Section

### Raw Materials

The PVDF powders (M_w_ = 530 000) were purchased from Sigma–Aldrich Co., Ltd. The PE wax (M_w_ = 2000‐3000) and N, N‐dimethylformamide (DMF) were obtained from Macklin and used as received. All the raw materials were directly used in experiments without any further purification.

### Preparation of PE wax/PVDF Films

First, the given amount of PVDF powders was dispersed homogeneously in 10 mL DMF by ultrasonication for 2.0 h at 40 °C. Then, the uniformly dispersed PE wax in acetone was added into the mixture and stirred vigorously at room temperature for 12.0 h. Subsequently, the suspension was cast into films on glass plates by an electric casting machine (AFA‐III, Hefei Ke Jing Materials Technology Company, Ltd.) at 60 °C. Next, the as‐cast films were repeatedly folded and hot pressed with a pressure of 35 MPa at 245 °C. Ultimately, the PE wax/PVDF films with different volume fractions (0%, 0.25%, 1.0%, 2.5%, 5.0%, and 10.0%) of PE wax were obtained. The thickness of final films prepared in this work was ≈10–15 µm.

### Characterizations

The crystalline phases and purity of the film were examined by X‐ray diffraction (XRD, Rigaku Smartlab SE) analysis. The morphologies and crystal structures of PE wax/PVDF film were observed by field emission scanning electron microscopy (FESEM, Zeiss Gemini 500) and polarized optical microscopy (Leica DM4). The phase structure is characterized by using Fourier transform infrared (FT‐IR, Bruker Vertex 80v) spectroscopy and confocal Raman microscopy (Renishaw inVia Reflex). The mechanical properties of the films were tested by electro mechanical universal testing machines (DMA, Jinan Testing Equipment WDW‐100). For electric, dielectric, and ferroelectric tests, gold electrodes (1.0 mm^2^) were first coated on two symmetric sides of the films. Dielectric data (capacitance, dielectric loss, and conductivity) were collected using an impedance analyzer (Agilent 4990A‐120) in a frequency region of 1 kHz to 10 MHz with applying 0.5 Vrms. Polarization and ferroelectric loops were measured on a ferroelectric test system (aixACCT TF3000). Charge–discharge (underdamped and overdamped) tests were executed on a commercial testing platform (CFD‐001, Gogo Instruments Technology, Shanghai, China), which incorporated a tailored RLC (resistance, inductance, and capacitance) load circuit.

## Conflict of Interest

The authors declare no conflict of interest.

## Supporting information



Supporting Information

## Data Availability

The data that support the findings of this study are available from the corresponding author upon reasonable request.
