# Supplementary material for: Free‐Volume Regulation Enables Significantly Enhanced Electrical Breakdown via Short‐Chain Molecular Spatial‐Positioning Intercalation into Poly(vinylidene fluoride)
Source: Adv Sci (Weinh). 2025 Nov 18;13(14):e17894. doi: 10.1002/advs.202517894 (PMC12970291; doi:10.1002/advs.202517894)
Supplement: Supplementary file 1 — Supporting Information [file ADVS-13-e17894-s001.docx]

Supporting Information

Free-volume regulation enables significantly enhanced electrical breakdown via short-chain molecular spatial-positioning intercalation into Poly(vinylidene fluoride)

Ziyue Wang, Jiyang Xie, Wanbiao Hu*

**Figure** **S1.** Crystal habit characterizations of the PE wax/PVDF film. (a) The polarized optical microscopy images, (b,c) the change of spherulite extinction vary with the polarized light.

Under orthogonal polarized light microscopy, the Maltese black cross, arising from the birefringence of spherulites, traverses the entire spherulite, which is caused by the unique birefringence characteristics of spherulites. Upon further observation of the spherulites using polarized light microscopy (Figures S1b,c), it is noted that the polarized light undergoes a rotation of 18 degrees. Consequently, the cross extinction pattern also changes correspondingly with this angle adjustment. This cross extinction pattern remains unchanged with stage rotation but varies with the rotation of polarized light, indicating an inherent feature of the spherulites.

**Figure S2.** Young’s Modulus of PE wax/PVDF Films.

The data reveal a substantial decrease in Young’s modulus from ~9.98 GPa for pure PVDF to ~0.11 GPa for the 10 vol% PE wax film. Crucially, the optimized composite with 2.5 vol% PE wax, which exhibits the highest breakdown strength (735 MV/m), shows a modulus of ~0.85 GPa—only marginally lower than pure PVDF (~9.98 GPa). Furthermore, the drastic modulus reduction in high-PE wax composites (*e.g.,* 10 vol%, modulus ≈0.11 GPa) does not correlate with breakdown strength trends. While the 10 vol% composite’s modulus approaches elastomeric levels, its breakdown strength plummets to ~419 MV/m—lower than pure PVDF. This divergence highlights that modulus reduction, rather than improvement, detrimentally impacts breakdown performance at high PE wax loadings, further dissociating modulus from the observed E_b_ enhancement.

In summary, the inverse correlation between Young’s modulus and breakdown strength in our PE wax/PVDF composites, combined with the electromechanical stability of high-modulus PVDF, definitively rules out mechanical modulus as a contributor to Eb enhancement. The data instead underscore free-volume engineering as the sole governing mechanism—a conclusion aligned with experimental observations, theoretical models, and the absence of electromechanical deformation signatures in in situ breakdown tests.


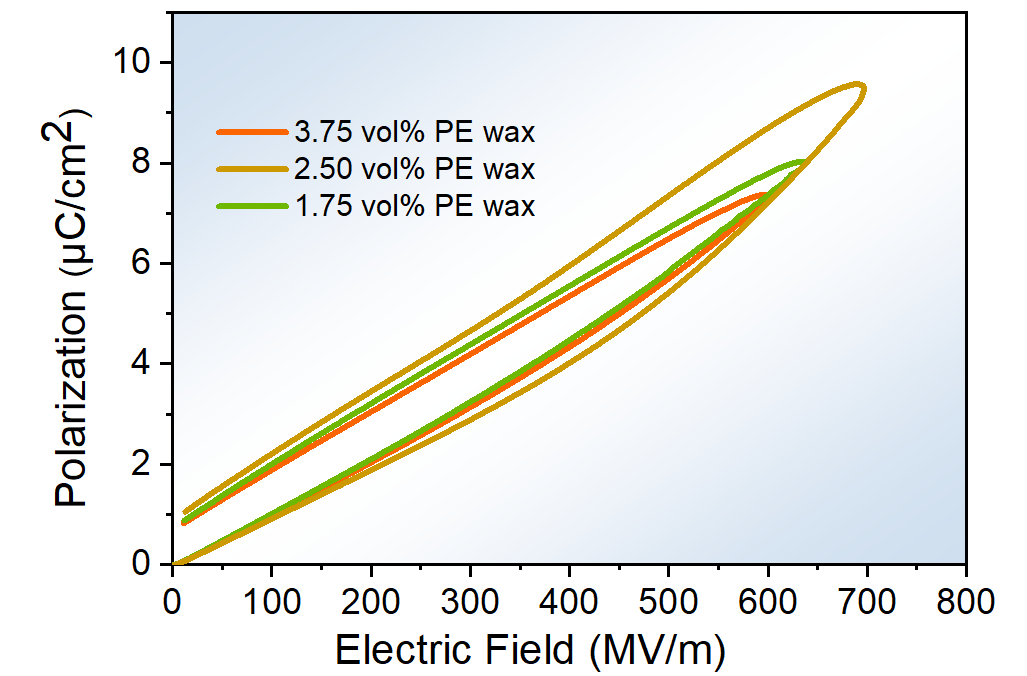


**Figure** **S3.** The corresponding P-E loop of PE wax/PVDF film with different PE content of 1.75 vol% and 3.75 vol%.

**Table S1.** Energy storage comparison of different fill percentages, especially 1.75 vol% and 3.75 vol%

|  | Pure PVDF | 0.25 vol% | 1.0 vol% | 2.5 vol% | 5.0 vol% | 10.0 vol% | 1.75 vol% | 3.75 vol% |
| --- | --- | --- | --- | --- | --- | --- | --- | --- |
| *U*_d_ (J/cm^3^) | 15.72 | 17.25 | 20.27 | 32.67 | 13.05 | 8.87 | **22.3** | **14.51** |
| *η* | 74.88% | 60.44% | 77.23% | 78.02% | 66.11% | 71.42% | **68.23 %** | **70.50 %** |

The comparison between other fill percentages, notably 1.75 vol% and 3.75 vol%, reveals energy densities of 22.3 J/cm³ and 14.51 J/cm³, respectively. This further reinforces the conclusion that the proximity to the optimal blending point is critical, as both formulations fall short of the performance exhibited by the 2.5 vol% sample. It is evident that the loop for the sample with 2.5 vol% of PE wax reveal a tighter hysteresis and greater polarization saturation. At this specific concentration, the energy density reaches its peak value of 32.67 J/cm³, coupled with an impressive efficiency of 78.02%. This result highlights that the composition at 2.5 vol% closely approximates PVDF’s free volume fraction, which is crucial for achieving optimal dielectric properties.


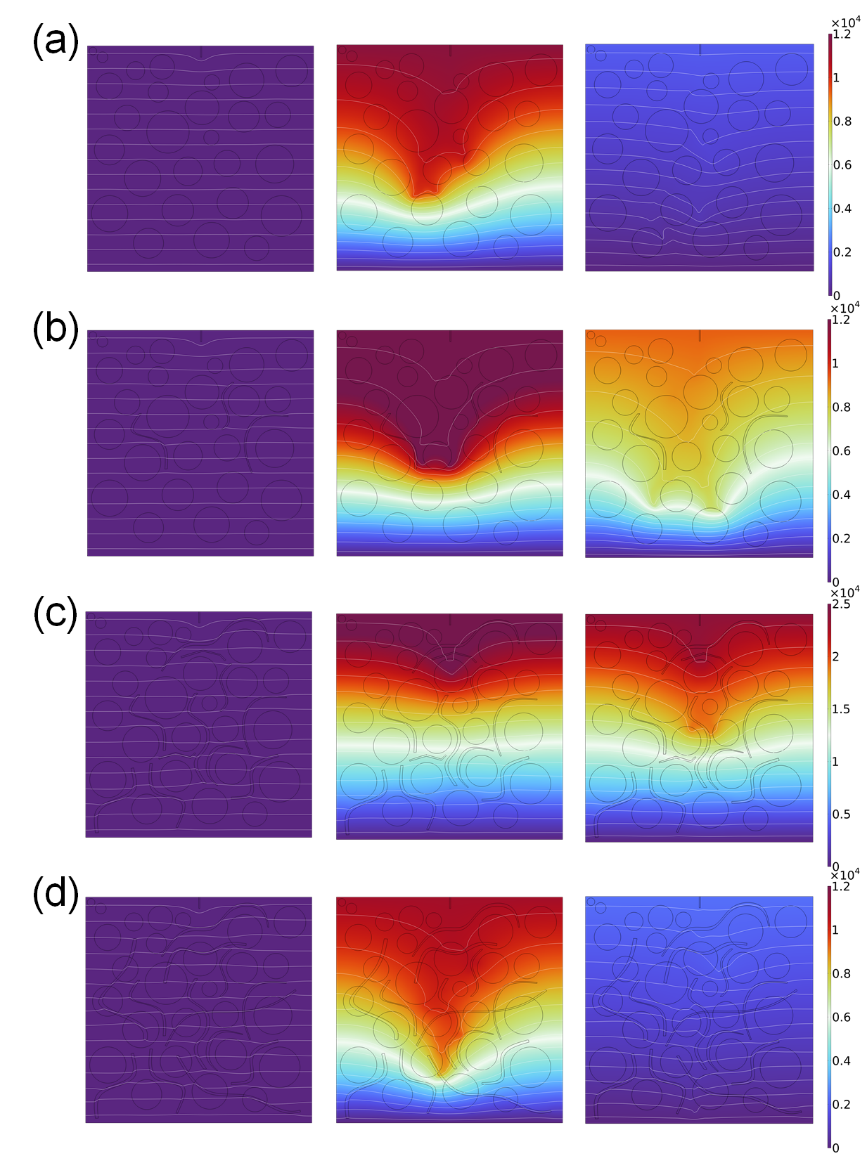


**Figure S4.** Electric potential evolution across PE wax/PVDF films.

Electric potential profiles for (a) 0 vol%, (b) 0.25 vol%, (c) 2.5 vol%, and (d) 5.0 vol% PE wax, illustrating charge trapping and interfacial polarization dynamics. Initial state shows steep potential gradients at amorphous regions, acting as charge traps that distort the electrostatic landscape. With optimal intercalation (2.5 vol%), potential gradients flatten as PE wax occupies free-volume, suppressing space charge accumulation. At overloaded content (10.0 vol%), localized potential wells reemerge near wax agglomerates, correlating with reduced breakdown strength due to conductive percolation pathways.


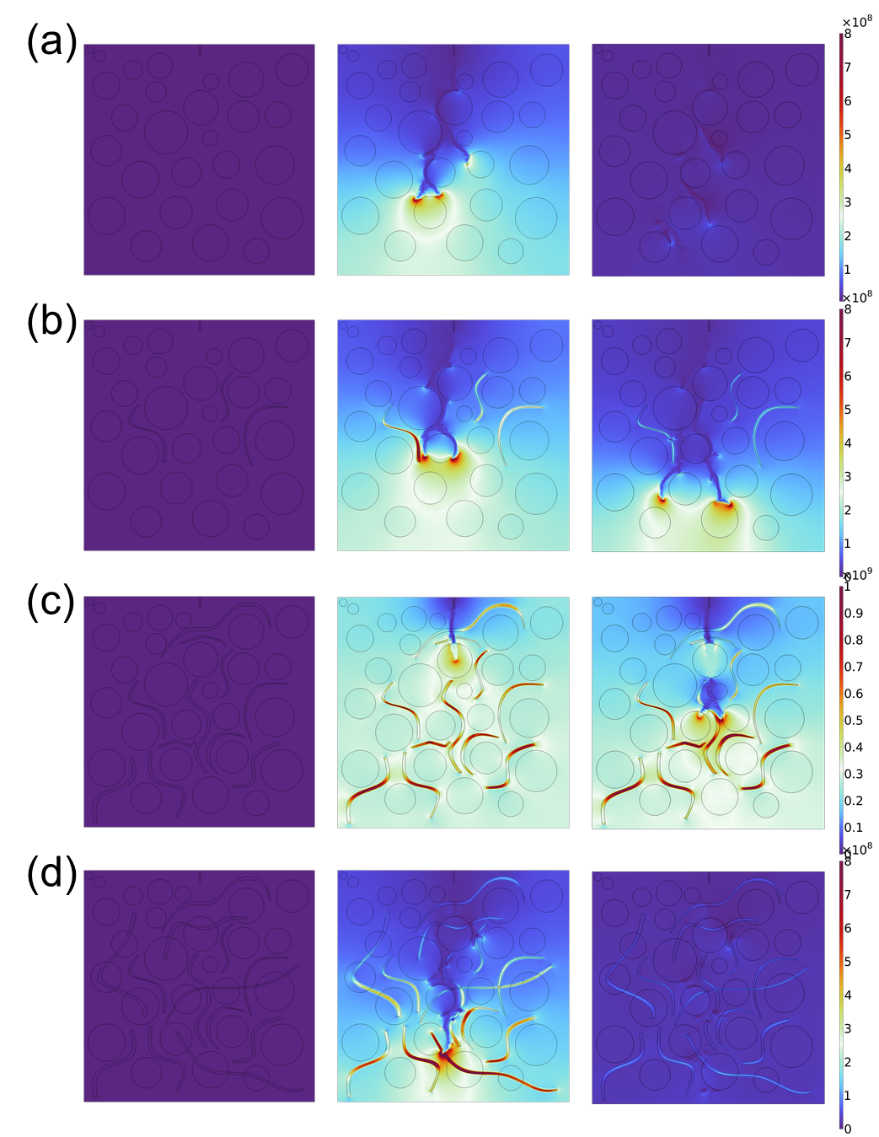


**Figure S5.** Electric field distribution in PE wax/PVDF films at varied intercalation levels.

Simulated electric field intensity maps of PVDF films with (a) 0 vol%, (b) 0.25 vol%, (c) 2.5 vol%, and (d) 5.0 vol% PE wax under an applied field. Arrows denote field directionality, and color gradients reflect localized field enhancement (red) or suppression (blue). In the initial state, the electric field concentrates at spherulite boundaries and amorphous-crystalline interfaces due to dielectric mismatch and free-volume voids. At the steady state (2.5 vol% PE wax), field homogenization occurs as PE wax fills inter-spherulitic gaps, reducing permittivity contrast and smoothing morphological discontinuities. Excessive PE wax (10.0 vol%) reintroduces field hotspots around agglomerated wax clusters, highlighting the criticality of optimal intercalation for field uniformity.

**Figure S6.** Electrical tree propagation dynamics in PE wax/PVDF films.

Time-resolved electrical tree growth in PE wax/PVDF films captured at 0.5 ns (initial), 1.0 ns (steady), and 1.5 ns (final) under a 735 MV/m field. In pristine PVDF, the tree propagates rapidly along free-volume pathways, forming dendritic branches by 1.5 ns. With 2.5 vol% PE wax, tree growth is fragmented and slowed, as intercalated wax obstructs free-volume channels, forcing tortuous paths. At 10.0 vol% PE wax, excessive wax induces heterogeneous regions where trees bifurcate around agglomerates, paradoxically accelerating late-stage failure despite initial suppression.
